# Pediatric Surgeons' Expectations of the EUPSA Global Surgery Committee Across World Bank Income Groups: A Two‐Round Modified Delphi Study

**DOI:** 10.1002/wjs.70541

**Published:** 2026-09-01

**Authors:** G. Brisighelli, G. T. Tekant, A. Gunnarsdóttir, A. R. Brohi, B. M. Basnet, C. M. Burgos, D. Di Fabrizio, E. Reuling, E. Stortelder, F. Pederiva, J. Lindert, J. Stoltz, M. Garriboli, M. Kakar, Ü. Celtik, U. Rolle

**Affiliations:** ^1^ Department of Paediatric Surgery School of Clinical Medicine Faculty of Health Sciences Johannesburg South Africa; ^2^ Department of Pediatric Surgery, Cerrahpaşa Faculty of Medicine Istanbul University‐Cerrahpaşa Istanbul Türkiye; ^3^ Department of Pediatric Surgery and Urology Karolinska University Hospital Astrid Lindgrens Childrens Hospital Stockholm Sweden; ^4^ Department of Women's and Children's Health Karolinska Institutet Stockholm Sweden; ^5^ Department of Pediatric Surgery Peoples University of Medical & Health Sciences (PUMHS) Nawabshah Sindh Pakistan; ^6^ Paediatric Laparoscopic/Thoracoscopic Surgery Shanghai China; ^7^ Department of Pediatric Surgery Kanti Children's Hospital Kathmandu Nepal; ^8^ Department of Pediatric Surgery Karolinska University Hospital Stockholm Sweden; ^9^ Department of Women's and Children's Health Karolinska Institutet Astrid Lindgrens Barnsjukhus Stockholm Sweden; ^10^ Pediatric Surgery Unit Salesi Children's Hospital Azienda Ospedaliero Universitaria delle Marche Ancona Italy; ^11^ Department of Pediatric Surgery Wilhelmina Children's Hospital University Medical Center Utrecht Utrecht the Netherlands; ^12^ Department of Pediatric Surgery Queen Fabiola Children's University Hospital Brussels Belgium; ^13^ Department of Pediatric Urology Wilhelmina Children's hosptial University Medical Center Utrecht Utrecht the Netherlands; ^14^ Department of Global Public Health and Bioethics Julius Center for Health Sciences and Primary Care University Medical Center Utrecht Utrecht the Netherlands; ^15^ Azienda Ospedaliera Universitaria Integrata (AOUI) Verona Italy; ^16^ Department of Paediatric Surgery University Medicine Rostock Rostock Germany; ^17^ Institute for Applied Health Sciences University of Applied Sciences Landshut Landshut Germany; ^18^ Department of General, Visceral and Minimally Invasive Surgery Hospital Mallersdorf Mallersdorf‐Pfaffenberg Germany; ^19^ Department of Paediatric Urology Evelina London Children's Hospital Guy's and St Thomas' NHS Foundation Trust London UK; ^20^ Department of Pediatric Surgery Riga Stradiņš University & Children's Clinical University Hospital Riga Latvia; ^21^ Department of Pediatric Surgery Ege University Faculty of Medicine, Bornova Izmir Turkey; ^22^ Department of Pediatric Surgery and Pediatric Urology University Hospital Frankfurt Goethe University Frankfurt am Main Germany

**Keywords:** capacity building, Delphi study, global surgery, low‐ and middle‐income countries, pediatric surgery, surgical training

## Abstract

**Aim:**

The European Pediatric Surgeons' Association (EUPSA) recently established a Global Surgery Committee to promote equitable access to pediatric surgical care through international collaboration and capacity building. This study aimed to explore and prioritize pediatric surgeons' expectations regarding the role and activities of the EUPSA Global Surgery Committee across different World Bank income groups, with particular attention to perspectives from low‐ and middle‐income settings.

**Methods:**

A two‐round modified Delphi study was conducted using online surveys distributed via EUPSA networks, pediatric surgical societies, and professional social media. In Round 1, participants provided up to five free‐text expectations; responses underwent inductive thematic analysis. In Round 2, participants ranked six themes, rated 20 subthemes on a 1–10 Likert scale and selected three top priorities. Countries were classified using the 2025 World Bank income groups, and descriptive statistics were used for analysis.

**Results:**

A total of 230 participants from 68 countries completed Round 1; 113 (49%) practiced in low‐ or lower‐middle‐income settings, 165 (72%) were consultant pediatric surgeons, and 196 (85%) worked in urban environments. Thematic analysis of 218 valid free‐text responses identified six themes spanning guidelines, training, research, advocacy, infrastructure, and cross‐cutting support. In Round 2, 65 complete responses were analyzed. Training‐related support ranked highest across all income groups, followed by guidelines and knowledge resources and by infrastructure and service delivery. Fellowships or observerships and structured mentorship were consistently among the highest‐rated subthemes. High‐income respondents more often prioritized guidelines and registry or multicenter research support, whereas low‐ and lower‐middle‐income respondents placed greater emphasis on equipment, infrastructure, and advocacy.

**Conclusions:**

Pediatric surgeons across income settings perceive EUPSA's global surgery role as multidimensional, with a consistent prioritization of capacity building through training, mentorship, guidelines, and systems‐oriented support.

## Graphical abstract

A two‐round modified Delphi study of paediatric surgeons from 68 countries identified six expectations of the EUPSA Global Surgery Committee. Across World Bank income groups, respondents prioritised sustainable capacity building through training and mentorship, supported by accessible guidelines, research collaboration, and context‐appropriate infrastructure and advocacy.

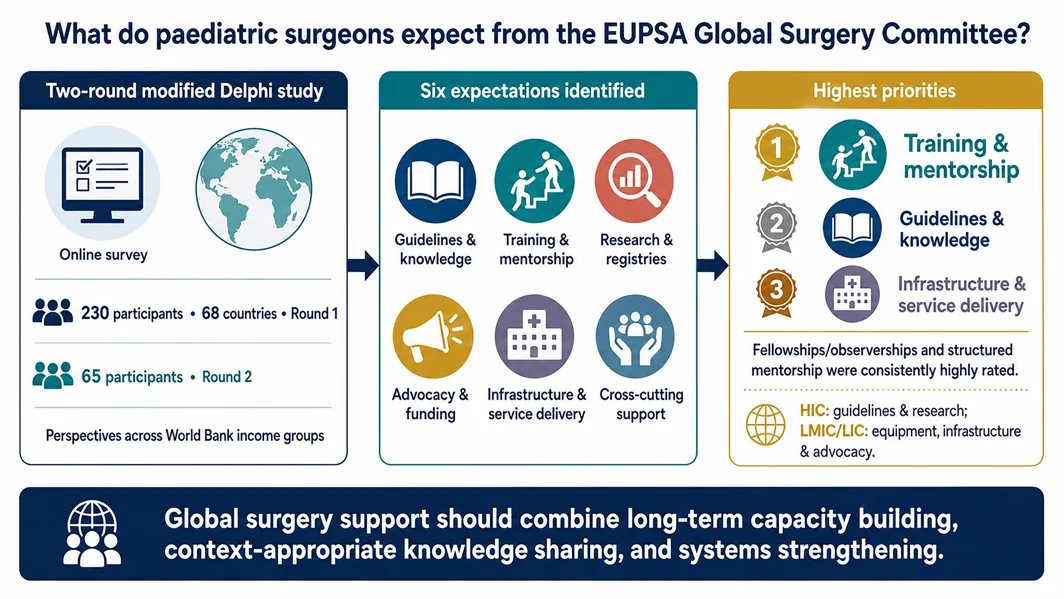

## 1 Introduction

Despite increasing global attention to surgical equity, substantial disparities persist in access to safe and timely pediatric surgical care across low‐ and middle‐income countries (LMICs) [1, 2]. Children in these settings face persistent inequities, including delayed diagnosis, limited access to specialist surgical care, and higher morbidity and mortality from otherwise treatable conditions [1, 3, 4]. Although global surgery initiatives have expanded over the past decade, structured engagement with stakeholders working in LMICs remains inconsistent, particularly within academic and professional surgical societies [5, 6].

Recent work, including the Global Initiative for Children's Surgery (GICS), has highlighted the importance of inclusive, bidirectional partnerships in global surgery, and emphasizing the need to move beyond unidirectional knowledge transfer toward collaborative models informed by local priorities and contexts [7, 8]. However, most existing frameworks have been developed from a public health or policy perspective, with relatively limited direct input from pediatric surgeons practicing in LMICs themselves [1]. As a result, there is a risk that well‐intentioned global surgical initiatives may not fully align with the expectations or needs of those they aim to support.

In response to these gaps, the European Pediatric Surgeons' Association (EUPSA) recently established a Global Surgery Committee with the aim of promoting equity in pediatric surgical care, fostering international collaboration, and supporting capacity development across diverse settings. As the Committee begins to define its strategic priorities and operational focus, it is essential that its activities are guided by the perspectives of pediatric surgeons working in LMICs.

Therefore, the aim of this study was to explore and identify, using a two‐round modified Delphi process, the expectations and priority areas for support from the EUPSA Global Surgery Committee, focusing on LMIC perspectives and comparison across World Bank income groups [9, 10, 11].

## 2 Methods

### 2.1 Study Design and Setting

This study used a modified Delphi methodology to explore and prioritize expectations regarding the role of the European Pediatric Surgeons' Association (EUPSA) Global Surgery Committee [9, 10]. An online multiround design was selected to enable structured input from geographically dispersed pediatric surgeons, pediatric urologists, and trainees working across low‐, middle‐, and high‐income settings. The study was conducted between September 2025 and April 2026.

### 2.2 Participants and Recruitment

Participants were recruited using purposive and snowball sampling through established international professional networks, including EUPSA‐affiliated mailing lists and WhatsApp groups, national and regional pediatric surgical societies, and professional social media platforms (LinkedIn, Instagram, and Facebook). The invitation contained a link to an anonymous online REDCap (Research Electronic Data Capture) survey and encouraged recipients to share the survey with eligible colleagues within their local and international professional networks. Eligible participants included pediatric surgeons, pediatric urologists, general surgeons with a pediatric surgery interest, trainees, and other clinicians involved in pediatric surgical care. Participation was voluntary and anonymous. Completion of the online survey constituted implied informed consent. No identifying information was collected within the survey; participants could optionally provide an email address via a separate, unlinked form to allow invitation to subsequent Delphi rounds. Data were collected using REDCap (Research Electronic Data Capture), hosted at the University of the Witwatersrand.

### 2.3 Round 1

The Round 1 questionnaire comprised seven items addressing participant demographics, professional characteristics, practice environment, and familiarity with the European Pediatric Surgeons' Association (EUPSA), followed by a single open‐ended question inviting participants to describe up to five expectations of the EUPSA Global Surgery Committee. Survey responses were screened for completeness and data integrity. Duplicate entries, identified based on identical demographic characteristics and overlapping free‐text responses, were excluded from all analyses. All remaining unique responses were included in the descriptive analysis of participant demographics, including country of practice, World Bank income classification, practice location, professional role, years of experience, sector of practice, and availability of key hospital resources.

Responses to the open‐ended question were analyzed using an inductive thematic analysis approach. Only responses containing meaningful free‐text content were included. One investigator (GB) performed the initial coding and developed a preliminary coding framework based on recurring concepts. This framework was subsequently reviewed by all investigators during a series of online meetings and refined through an iterative process of discussion and majority agreement. Areas of disagreement were resolved through further discussion and, when necessary, majority voting. During this consensus process, an additional theme of general cross‐cutting support was introduced to capture broad or nonspecific expectations, whereas the themes relating to educational resources and infrastructure were refined to distinguish nonphysical and physical forms of support, respectively. The final coding framework comprised six overarching themes encompassing 20 subthemes, which informed the development of the structured Round 2 questionnaire.

### 2.4 Round 2

The Round 2 questionnaire was developed directly from the thematic framework generated during Round 1 and was designed to quantify and prioritize participants' expectations of the EUPSA Global Surgery Committee. Only participants who had voluntarily provided an email address through a separate, unlinked form during Round 1 were invited to participate.

The questionnaire consisted of four sections. First, participants confirmed their current professional role. Second, they were asked to rank the six overarching themes identified during Round 1 from 1 (highest priority) to 6 (lowest priority). Third, participants rated the importance of each of the 20 subthemes using a 10‐point Likert scale (1 = not at all important and 10 = extremely important). Finally, participants were asked to select up to three subthemes from the list of 20 that they considered their highest priorities (“top three priorities” question) of the EUPSA Global Surgery Committee and could provide optional comments on whether the themes adequately reflected the needs and priorities of their local context.

### 2.5 Statistical Analysis

Descriptive statistics were used to summarize participant characteristics and survey responses. Countries were classified according to the World Bank income classification for 2025 [11]. Quantitative results were supplemented by a qualitative review of free‐text comments to aid interpretation.

A third Delphi round was not undertaken because the objective of the study was to explore and prioritize participant‐generated expectations rather than to achieve formal consensus on a predefined set of statements. Following Round 2, a stable and consistent pattern of priorities was observed across World Bank income groups, and the investigator group considered that additional rounds were unlikely to substantially alter the overall findings.

The Round 1 and Round 2 survey instruments are provided as Supplementary Material.

## 3 Results

### 3.1 Round 1

A total of 240 individuals participated in Round 1, of whom 177 provided an email address. After exclusion of duplicate entries, 230 unique participants were included in the analysis. Table 1 summarizes participants' country of origin according to the 2025 World Bank income classification, whereas Table 2 outlines participants' demographics and professional characteristics. Nearly half of the respondents were based in low‐ or lower‐middle‐income countries, most were consultant pediatric surgeons (72%, *n* = 165), and the majority worked in urban settings (85%, *n* = 196), with a wide range of years of professional experience.

**TABLE 1:** Country of origin of participants in Rounds 1 and 2 and country classification (according to World Bank income group—2025).

Income group/Country of origin	Round 1 *n* (%)	Round 2 *n* (%)
Low income countries (LIC)	21 (9.13%)	5 (7.7%)
Uganda	4 (1.74%)	1 (1.5%)
Democratic republic of Congo	4 (1.74%)	—
Liberia	3 (1.30%)	2 (3.1%)
Gambia	2 (0.87%)	1 (1.5%)
Sudan	2 (0.87%)	—
Ethiopia	2 (0.87%)	1 (1.5%)
Malawi	1 (0.43%)	—
Benin	1 (0.43%)	—
Rwanda	1 (0.43%)	—
Sierra Leone	1 (0.43%)	—
Lower middle‐income countries (LMIC)	81 (35.22%)	22 (33.8%)
Pakistan	18 (7.83%)	3 (4.6%)
Nigeria	12 (5.22%)	6 (9.2%)
Tanzania	8 (3.48%)	—
India	7 (3.04%)	5 (7.7%)
Cameroon	5 (2.17%)	—
Morocco	4 (1.74%)	—
Egypt	4 (1.74%)	1 (1.5%)
Ghana	4 (1.74%)	—
Nepal	4 (1.74%)	2 (3.1%)
Senegal	3 (1.30%)	1 (1.5%)
Kenya	3 (1.30%)	2 (3.1%)
Côte d’Ivoire	2 (0.87%)	—
Zimbabwe	1 (0.43%)	—
Nicaragua	1 (0.43%)	—
Zambia	1 (0.43%)	—
Tunisia	1 (0.43%)	1 (1.5%)
Myanmar	1 (0.43%)	1 (1.5%)
Philippines	1 (0.43%)	—
Lebanon	1 (0.43%)	—
Upper middle‐income countries (UMIC)	57 (24.78%)	15 (23.1%)
Mexico	24 (10.43%)	7 (10.8%)
Turkey	9 (3.91%)	1 (1.5%)
South Africa	4 (1.74%)	1 (1.5%)
Iraq	4 (1.74%)	—
Brazil	3 (1.30%)	—
Argentina	3 (1.30%)	2 (3.1%)
Algeria	2 (0.87%)	—
Dominican Republic	2 (0.87%)	1 (1.5%)
Ecuador	2 (0.87%)	1 (1.5%)
Belize	1 (0.43%)	—
Thailand	1 (0.43%)	1 (1.5%)
Venezuela	1 (0.43%)	—
Peru	1 (0.43%)	—
High income countries (HIC)	71 (30.87%)	23 (35.4%)
Italy	10 (4.35%)	5 (7.7%)
Germany	8 (3.48%)	2 (3.1%)
Sweden	5 (2.17%)	4 (6.2%)
Portugal	4 (1.74%)	—
Netherlands	4 (1.74%)	1 (1.5%)
Belgium	3 (1.30%)	2 (3.1%)
Switzerland	3 (1.30%)	—
Latvia	3 (1.30%)	1 (1.5%)
United Kingdom	3 (1.30%)	—
Poland	3 (1.30%)	1 (1.5%)
Saudi Arabia	3 (1.30%)	—
Spain	2 (0.87%)	—
Slovenia	2 (0.87%)	1 (1.5%)
France	2 (0.87%)	1 (1.5%)
Norway	2 (0.87%)	1 (1.5%)
Oman	2 (0.87%)	1 (1.5%)
Russia	1 (0.43%)	—
Qatar	1 (0.43%)	—
Canada	1 (0.43%)	—
Bahrain	1 (0.43%)	—
Slovakia	1 (0.43%)	—
United Arab Emirates	1 (0.43%)	—
Australia	1 (0.43%)	—
USA	1 (0.43%)	—
Austria	1 (0.43%)	1 (1.5%)
Israel	1 (0.43%)	—
Guyana	1 (0.43%)	1 (1.5%)
Costa Rica	1 (0.43%)	—
Grand total	230 (100%)	65 (100%)

**TABLE 2:** Demographic and professional characteristics of Round 1 participants.

	Total	%
Practice location		
Urban/City	196	85.22%
Both urban and rural practice	21	9.13%
Rural	13	5.65%
Total	230	100.00%
Current position/role		
Consultant/Specialist pediatric surgeon	165	71.74%
Pediatric surgery/Pediatric urology trainee/Registrar	23	10.00%
General surgeon with pediatric surgery interest	17	7.39%
Fellow (pediatric surgery/Urology)	8	3.48%
Medical officer or junior doctor	7	3.04%
Consultant/Specialist pediatric urologist	5	2.17%
Other: (Pediatric anesthesiologist/ICU doctor)	5	2.17%
Total	230	100.00%
Years of experience		
4–10 years	86	37.39%
More than 20 years	59	25.65%
11–20 years	57	24.78%
1–3 years	23	10.00%
Less than 1 year	5	2.17%
Total	230	100.00%
Available resources^a^		
Dedicated pediatric operating room(s)	181	78%
Dedicated anesthesia service (regular)	167	72.61%
Neonatal/Pediatric intensive care unit (NICU/PICU)	176	76.52%
Dedicated pediatric surgical team/Unit	193	83.91%
Regular access to imaging (CT/MRI/Ultrasound)	183	79.57%
None of the above	4	1.74%
Unsure	1	0.43%
Practice sector^a^		
Public sector	150	65.22%
Private sector	70	30.43%
Academic/University hospital	105	45.65%
Nonprofit/Nongovernmental organization/Mission hospital	13	5.65%
Other (free text: camps)	1	0.43%
Familiarity with EUPSA		
No, I have never attended a EUPSA conference, but I am familiar with EUPSA	85	36.96%
No, I have never attended a EUPSA conference, and I am not familiar with EUPSA	64	27.83%
I am a EUPSA member	44	19.13%
I am not a EUPSA member, but I have attended a EUPSA conference	37	16.09%
Total	230	100.00%

^a^ Percentages do not sum to 100% as multiple responses were allowed.

For the qualitative analysis of expectations (Question 8), nine responses were excluded because the question was left blank, and three were excluded because an email address was provided instead of an expectation. Therefore, a total of 218 responses were considered valid and included in the thematic analysis.

An inductive thematic analysis of 218 valid free‐text responses identified six overarching themes: (1) guidelines, knowledge, and educational resources; (2) training, mentorship, fellowships, and exchanges; (3) research, registries, and publications; (4) advocacy, awareness, workforce, and funding; (5) infrastructure, equipment, telemedicine, and service delivery; and (6) general or cross‐cutting support. Together, these comprised 20 associated subthemes and formed the conceptual framework for the Round 2 questionnaire (Figure 1).

**FIGURE 1:**
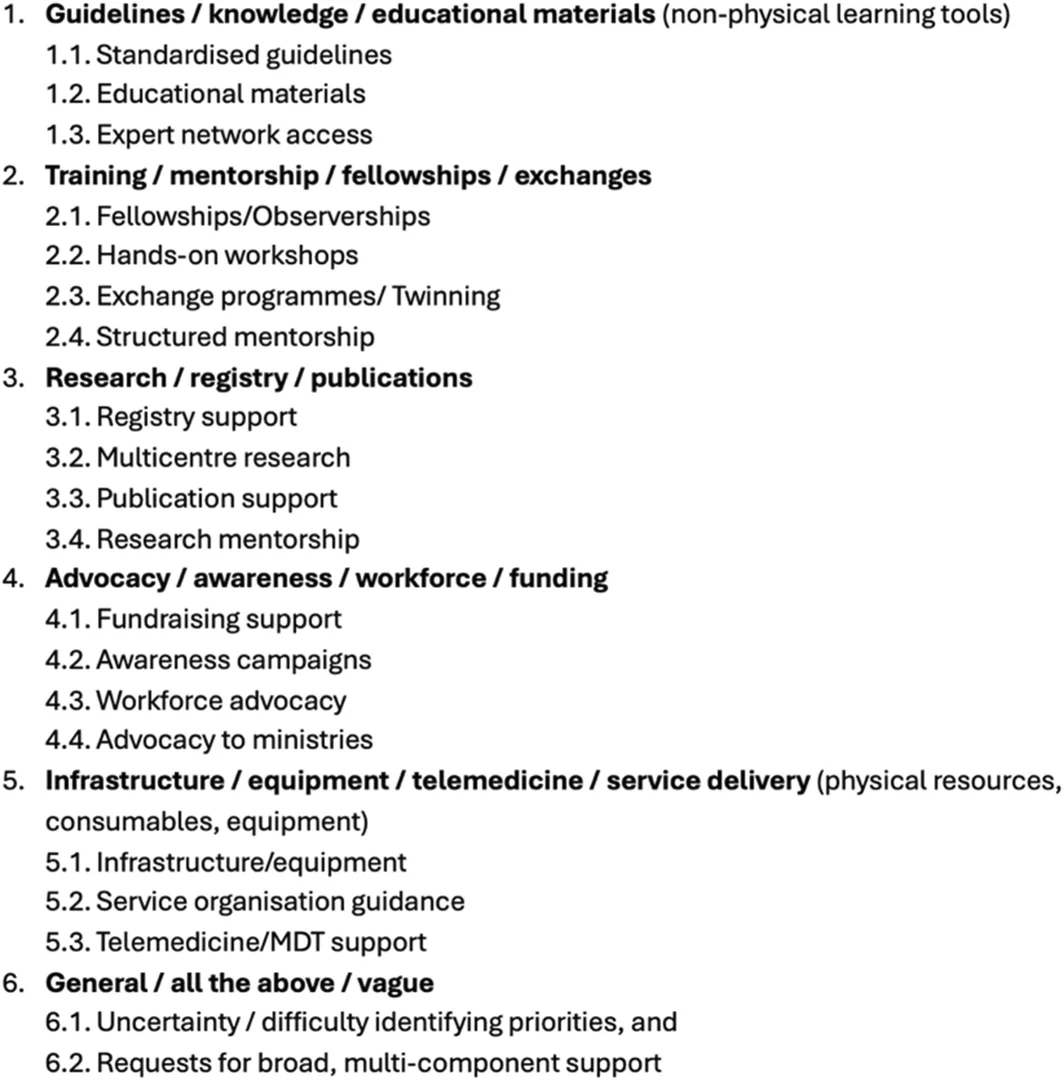
Inductive thematic framework of expectations of the EUPSA Global Surgery Committee derived from Round‐1 (Question 8) free‐text responses, showing six overarching themes and associated subthemes.

Participants reported a mean of 2.3 themes per response (range 1–6) and a mean of 3.8 subthemes per response (range 0–15).

Participants described a broad expectation that the EUPSA Global Surgery Committee should promote sustainable partnerships extending beyond isolated educational activities. Training and mentorship requests extended beyond fellowships and observerships to include longitudinal mentorship, hands‐on workshops, and structured exchange programs. Respondents also emphasized the need for accessible and context‐appropriate educational resources, including open‐access guidelines, webinars, and expert networks.

Research‐related expectations focused on strengthening local research capacity through multicenter registries, collaborative projects, mentorship in study design and data analysis, and support for publications and funding opportunities. Infrastructure requests included access to pediatric‐specific equipment, minimally invasive surgery resources, anesthetic and intensive care support, and practical guidance for service development. Finally, many participants highlighted the importance of advocacy, particularly to improve recognition of pediatric surgery, strengthen workforce development, and facilitate equitable collaboration between high‐ and low‐resource settings. Although the same broad themes emerged across all income groups, respondents from lower‐resource settings more frequently linked training opportunities to infrastructure and workforce limitations, whereas participants from higher‐income settings more commonly emphasized collaborative research and registry development.

### 3.2 Round 2

Of 74 responses received in Round 2, 9 (12.2%) were excluded due to incomplete rankings, leaving 65 complete responses for analysis. Participants represented 33 countries across all World Bank income classifications, including 23 (35.4%) from HICs, 22 (33.8%) from LMICs, 15 (23.1%) from UMICs, and 5 (7.7%) from LICs (Table 1). Participants' roles were consultant/specialist pediatric surgeon (*n* = 48, 73.8%), consultant/specialist pediatric urologist (*n* = 1, 1.5%), pediatric surgery/pediatric urology trainee/registrar (*n* = 7, 10.8%), general surgeon with pediatric surgery interest (*n* = 4, 6.2%), Fellow in pediatric surgery/urology (*n* = 2, 3.1%), and “Other” (*n* = 3, 4.6%, all 3 being pediatric anesthesiologists). Exclusions were nonsystematic by country, income group, or professional role.

Across all respondents, training, mentorship, fellowships, and exchanges received the highest priority, followed closely by guidelines and knowledge resources, and by infrastructure and service organization (Figure 2). At the income‐group level, training support ranked first in LMIC, UMIC, and LIC respondents, whereas HIC respondents gave slightly higher priority to guidelines and knowledge resources, with training a close second. Despite these modest differences in ordering, the same three domains—training, guidelines, and infrastructure—consistently occupied the top tier across all income groups.

**FIGURE 2:**
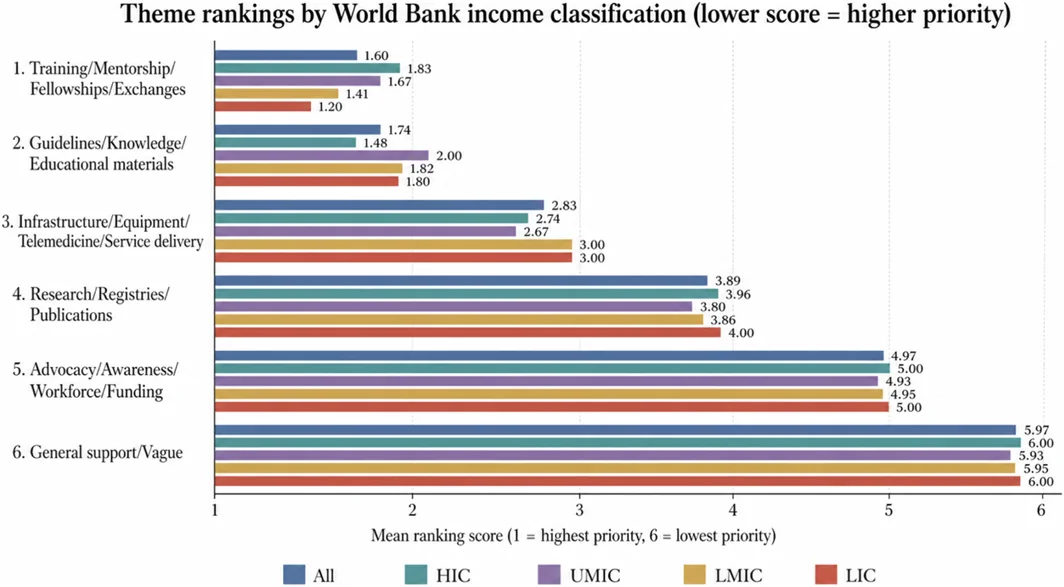
Theme rankings by World Bank income classification in Round 2 (*n* = 65). Participants ranked six themes from 1 (highest priority) to 6 (lowest priority). Values are mean ranks. Standard deviations are provided in Table 3. Sample sizes: HIC *n* = 23, UMIC *n* = 15, LMIC *n* = 22, and LIC *n* = 5.

Importance ratings for the 20 subthemes showed a broadly similar hierarchy (Table 3). The highest‐rated subthemes overall were standardized clinical guidelines and protocols, infrastructure and equipment, fellowships or observerships, multicenter research, hands‐on workshops, exchange or twinning programs, and structured mentorship, all with mean ratings ≥ 8.4 on the 1–10 Likert scale. Although mean scores varied slightly by the income group, the same cluster of training, guideline, infrastructure, and research subthemes consistently occupied the top of the distribution across HIC, UMIC, LMIC, and LIC respondents. LMIC and LIC respondents tended to give relatively higher ratings to advocacy‐focused items, such as ministries advocacy and fundraising, whereas HIC and UMIC respondents more often prioritized registry and multicenter research support within the research domain.

**TABLE 3:** Importance ratings for 20 subthemes, grouped under the main themes of training, guidelines and knowledge resources, research and registries, infrastructure and service organization, and advocacy/general support, by World Bank income classification in Round 2 (*n* = 65).

Rank	Subtheme	All mean (SD)	HIC mean (SD)	UMIC mean (SD)	LMIC mean (SD)	LIC mean (SD)
Guidelines and knowledge subthemes						
1	Guidelines—Standardized protocols	8.5 (2.2)	9.0 (1.1)	7.9 (2.7)	8.6 (2.5)	7.8 (2.9)
8	Guidelines—Expert networks	8.3 (2.1)	8.8 (1.4)	8.1 (2.7)	7.8 (2.4)	8.8 (1.6)
10	Guidelines—Educational materials	8.2 (2.2)	8.7 (1.3)	8.1 (2.5)	8.0 (2.7)	8.0 (3.1)
Infrastructure and equipment subthemes						
2	Infrastructure—Equipment	8.5 (2.1)	8.4 (1.6)	8.1 (2.3)	8.8 (2.2)	8.6 (3.1)
12	Infrastructure—Service organization	8.2 (2.1)	8.2 (1.4)	7.5 (2.9)	8.6 (2.1)	8.4 (2.5)
17	Infrastructure—Telemedicine/MDT	7.7 (2.1)	8.0 (1.6)	7.1 (2.6)	7.8 (2.0)	7.8 (2.9)
Training and mentorship subthemes						
3	Training—Fellowships/observerships	8.4 (2.1)	8.5 (1.5)	7.8 (2.7)	8.8 (2.0)	8.4 (2.6)
5	Training—Hands‐on workshops	8.4 (2.1)	8.2 (1.7)	7.7 (2.5)	8.9 (2.0)	9.0 (2.2)
6	Training—Exchange/twinning	8.4 (2.2)	8.5 (1.6)	7.5 (3.1)	8.8 (1.8)	8.6 (3.1)
7	Training—Structured mentorship	8.4 (2.1)	8.0 (2.2)	8.3 (2.4)	8.5 (1.8)	9.8 (0.4)
Research and registries subthemes						
4	Research—Multicenter research	8.4 (2.1)	8.5 (1.7)	8.3 (2.7)	8.5 (2.1)	8.6 (1.9)
9	Research—Registry/database	8.3 (2.3)	8.4 (1.9)	8.0 (3.0)	8.3 (2.2)	8.2 (3.5)
11	Research—Publication support	8.2 (2.1)	7.8 (2.0)	8.5 (2.3)	8.4 (2.1)	8.8 (1.8)
14	Research—Research mentorship	8.0 (2.1)	8.1 (1.6)	8.0 (2.6)	8.0 (2.1)	8.0 (2.7)
Advocacy and awareness subthemes						
13	Advocacy—Ministries advocacy	8.0 (2.5)	7.9 (1.9)	6.9 (3.7)	8.8 (1.8)	8.8 (2.7)
15	Advocacy—Workforce advocacy	7.9 (2.4)	7.5 (1.9)	7.4 (3.0)	8.5 (2.1)	8.2 (3.5)
16	Advocacy—Awareness campaigns	7.8 (2.3)	7.9 (1.8)	6.8 (3.1)	8.1 (2.1)	8.4 (2.5)
18	Advocacy—Fundraising	7.5 (2.6)	7.1 (2.2)	6.9 (3.1)	8.0 (2.2)	8.4 (3.6)
General support subthemes						
19	General—Comprehensive support	7.5 (1.9)	7.2 (1.6)	6.8 (2.2)	7.8 (2.0)	9.2 (1.3)
20	General—Priority identification	7.4 (2.0)	7.0 (1.5)	6.9 (2.5)	8.0 (2.0)	8.8 (2.2)

*Note:* Participants rated each subtheme on a 1–10 Likert scale (1 = not at all important, 10 = extremely important). Values are mean (standard deviation). Sample sizes: HIC *n* = 23, UMIC *n* = 15, LMIC *n* = 22, LIC *n* = 5.

When restricted to selecting only three top priorities from the 20 subthemes, participants most frequently chose standardized clinical guidelines and protocols, registry/database support, exchange or twinning programs, and equipment and physical infrastructure, with multicenter collaborative research and hands‐on workshops also commonly selected (Figure 3). These priorities were broadly shared across income groups, although LMIC and LIC respondents more often highlighted equipment and physical infrastructure, whereas HIC and UMIC respondents more commonly prioritized registry/database and multicenter research support, with hands‐on workshops featuring prominently in UMIC settings.

**FIGURE 3:**
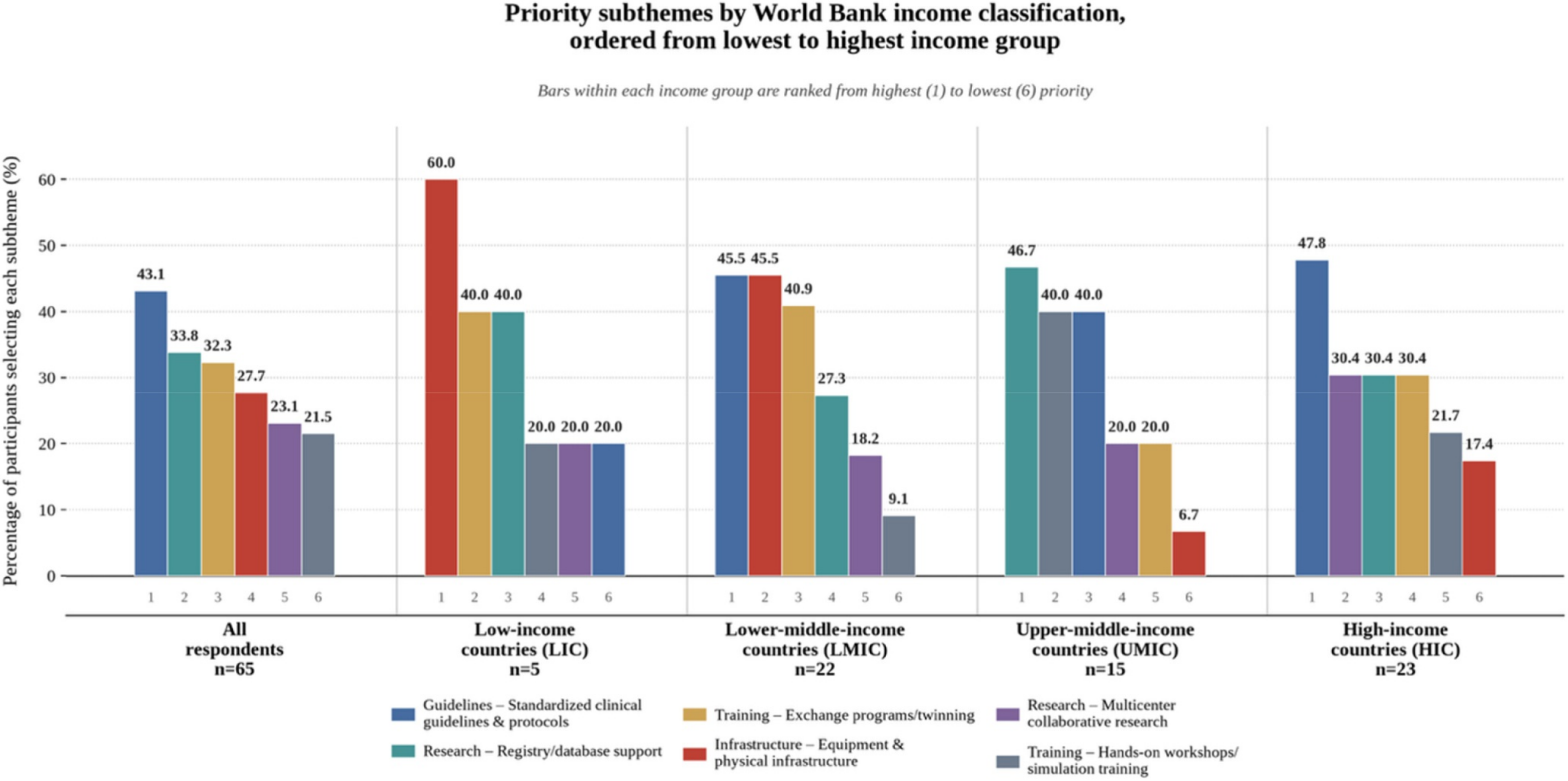
Top‐three priority selections for key subthemes by World Bank income classification in Round 2 (*n* = 65). Participants could select up to three subthemes as their highest priorities from the list of 20 subthemes presented in Round 2. The figure shows subthemes selected by at least 14 participants overall, with percentages calculated for all participants and for each income group (HIC, UMIC, LMIC, and LIC). Within each income group, subthemes are ordered from the highest (rank 1) to the lowest (rank 6) percentage of participants selecting that subtheme, so that shifts in priority patterns across income levels can be seen directly along the horizontal axis.

Of the 65 respondents in Round 2, 62 felt the list adequately reflected the needs in their context; the three who disagreed argued that many items already exist without improving training or outcomes, that some items overlapped, and that support structures were lacking in their settings.

## 4 Discussion

This two‐round modified Delphi study describes how pediatric surgeons across World Bank income groups conceptualize the role of a regional pediatric surgical society in global surgery and what they expect from the newly formed EUPSA Global Surgery Committee. Our findings demonstrate that all subthemes were rated highly across respondents, reflecting a broad perception that multiple domains are important for global surgery engagement. Within this overall pattern, a relative hierarchy of priorities was observed, with the highest‐rated subthemes concentrated in training, guidelines, infrastructure, and research. Four of the ten highest‐rated subthemes were training‐related, underscoring that capacity building through fellowships or observerships, hands‐on workshops, exchanges, and structured mentorship is viewed as a core expectation of a regional society. This aligns with previous reports that surgeons in low‐ and middle‐income settings often identify skills development, longitudinal mentorship, and opportunities for bidirectional exchanges as among the most tangible and sustainable benefits of international collaboration [5, 7].

The prominence of fellowships/observerships and structured mentorship across all income groups suggests an appetite for longitudinal relationship‐based capacity building. Consistent with calls for bidirectional global surgery partnerships, such programs need not be limited to LMIC‐to‐HIC observerships, but can include exchanges within and between LMICs, where practice environments and resource constraints are more comparable [5, 8, 12]. Hands‐on workshops, exchanges, and twinning were also highly rated, particularly by respondents from LMIC, LIC, and UMIC settings, which are often characterized by high clinical volume and constrained resources. For the committee, this implies that training initiatives must also be tailored to the realities of host settings, ensuring that acquired skills are immediately applicable and not dependent on unavailable technologies [12].

Respondents' emphasis on standardized guidelines, infrastructure and equipment, registries, and longitudinal training suggests that support perceived as most valuable is that which strengthens local systems and persists beyond short‐term visits [3, 4, 8]. This aligns with concerns that mission‐style outreach has limited long‐term impact unless embedded within sustained capacity‐building frameworks that include co‐developed protocols, training, and local ownership [12, 13]. For the EUPSA Global Surgery Committee, this suggests the need for integrated strategies linking guideline development, training, infrastructure planning, and collaborative research [5, 8]. In practice, this could mean co‐developing regionally adapted protocols with LMIC partners, piloting them through fellowships and workshops, and using registry data to iteratively refine both content and delivery [3, 14, 15].

A notable feature of our results is the high degree of convergence across income groups. Training, guidelines, and infrastructure consistently formed the top tier of priorities across all respondents. Within this overall agreement, differences in emphasis were observed. High‐income respondents more frequently prioritized guidelines and research infrastructure, including registries and multicenter studies, whereas LMIC and LIC respondents placed greater emphasis on equipment, infrastructure, and advocacy. These differences likely reflect variations in baseline resources and available levers for change, with lower‐income settings continuing to face fundamental constraints in infrastructure and financing [2, 3, 4]. For the committee, this highlights the need to pair cross‐cutting initiatives, such as guideline and registry development, with targeted support that addresses context‐specific bottlenecks in lower‐resource environments [5, 8].

This study has several limitations. First, the open‐ended Round‐1 question included example domains, which may have introduced a degree of priming and influenced how participants framed their responses; however, the use of prompts to guide responses is an accepted approach in Delphi methodology [9, 10]. Second, participation was voluntary and may have attracted respondents with a preexisting interest in global surgery and included in various international context, potentially limiting representativeness. Third, although respondents were drawn from 68 countries across all World Bank income groups, participants from low‐income countries (LICs) were relatively underrepresented, particularly in Round 2. Although respondents from LMIC and LIC settings together comprised a substantial proportion of the cohort, the lower participation from LICs may limit the extent to which the findings fully capture the priorities of surgeons working in the most resource‐constrained settings. Finally, some regions and subspecialties may remain underrepresented.

Despite these limitations, this study provides a large, diverse, and directly elicited set of perspectives from pediatric surgeons across income settings. By systematically identifying and prioritizing expectations, it offers an empirical foundation to guide the development of the EUPSA Global Surgery Committee.

## 5 Conclusions

In this two‐round modified Delphi study, pediatric surgeons from across income settings identified training, standardized guidelines, infrastructure, and collaborative research as the most important areas in which a regional pediatric surgical society can support global surgery efforts. Despite diverse practice contexts, there was substantial convergence in priorities across World Bank income groups, supporting a model of global engagement that emphasizes shared agendas and mutual learning rather than unidirectional assistance. For the EUPSA Global Surgery Committee, these findings provide a pragmatic framework for early strategic planning, highlighting the value of focusing on structured fellowships and mentorship, context‐appropriate guideline development, and data‐driven initiatives—such as registries and multicenter research—that can underpin advocacy and long‐term capacity strengthening.

## Author Contributions

**G. Brisighelli:** conceptualization, data curation, formal analysis, writing – original draft, methodology, investigation, writing – review and editing. **G. T. Tekant:** conceptualization, supervision, writing – review and editing, methodology. **A. Gunnarsdóttir:** conceptualization, supervision, writing – review and editing. **A. R. Brohi:** conceptualization, supervision, writing – review and editing. **B. M. Basnet:** conceptualization, supervision, writing – review and editing. **C. M. Burgos:** conceptualization, supervision, writing – review and editing, methodology. **D. Di Fabrizio:** conceptualization, supervision, writing – review and editing. **E. Reuling:** conceptualization, writing – review and editing, supervision. **E. Stortelder:** conceptualization, supervision, writing – review and editing. **F. Pederiva:** conceptualization, supervision, writing – review and editing. **J. Lindert:** conceptualization, supervision, writing – review and editing. **J. Stoltz:** conceptualization, supervision, writing – review and editing. **M. Garriboli:** conceptualization, supervision, writing – review and editing. **M. Kakar:** conceptualization, supervision, writing – review and editing. **Ü. Celtik:** conceptualization, writing – review and editing, supervision. **U. Rolle:** conceptualization, supervision, writing – review and editing.

## Funding

The authors have nothing to report.

## Ethics Statement

All authors comply with the journal's ethical policies. This study was approved by the University of the Witwatersrand Human Research Ethics Committee (Medical) (Certificate: M260131).

## Consent

Informed consent was obtained from all individual participants included in the study. Participation was voluntary and anonymous, and completion of the survey constituted implied consent.

## Conflicts of Interest

The authors declare no conflicts of interest.

## Supporting information


Supporting Information S1

Supporting Information S2

## Data Availability

The data that support the findings of this study are available from the corresponding author upon reasonable request.

## References

[1] E. Bryce , M. Fedatto , and D. Cunningham , “Providing Paediatric Surgery in Low‐Resource Countries,” Bmjpo 7, no. 1 (2023): e001603, 10.1136/bmjpo-2022-001603. PMC9923288 36764702

[2] K. Landrum , C. F. Cotache‐Condor , Y. Liu , et al., “Global and Regional Overview of the Inclusion of Paediatric Surgery in the National Health Plans of 124 Countries: An Ecological Study,” BMJ Open 11, no. 6 (2021): e045981, 10.1136/bmjopen-2020-045981. PMC8211076 34135040

[3] N. J. Wright , A. J. M. Leather , N. Ade‐Ajayi , et al., “Mortality From Gastrointestinal Congenital Anomalies at 264 Hospitals in 74 Low‐Income, Middle‐Income, and High‐Income Countries: A Multicentre, International, Prospective Cohort Study,” Lancet 398, no. 10297 (2021): 325–339, 10.1016/S0140-6736(21)00767-4. PMC8314066 34270932

[4] A. G. Sykes , J. Seyi‐Olajide , E. A. Ameh , et al., “Estimates of Treatable Deaths Within the First 20 Years of Life From Scaling up Surgical Care at First‐Level Hospitals in Low‐ and Middle‐Income Countries,” World Journal of Surgery 46, no. 9 (2022): 2114–2122, 10.1007/s00268-022-06622-w. PMC9334432 35771254

[5] S. Bandyopadhyay , G. S. Philipo , T. Banu , Z. Bokhary , and K. Lakhoo , “Global Initiative for Children’s Surgery (GICS): A Decade in Review,” World Journal of Pediatric Surgery 8, no. 4 (2025): e001084, 10.1136/wjps-2025-001084. PMC12496102 41050908

[6] S. L. M. Greenberg , H. C. Cockrell , G. Hyman , L. Goodman , N. Kaseje , and K. T. Oldham , “The Global Initiative for Children’s Surgery: Conception, Gestation, and Delivery,” Pediatric Surgery International 39, no. 1 (2022): 48, 10.1007/s00383-022-05319-4. PMC9744037 36507955

[7] M. U. Khalid , A. Mac , M. Biderman , L. Errett , and A. Sriharan , “Partnering to Build Surgical Capacity in Low‐Resource Settings: A Qualitative Study of Canadian Global Surgeons,” BMJ Open 13, no. 3 (2023): e070148, 10.1136/bmjopen-2022-070148. PMC10040043 36948558

[8] Global Initiative for Children’s Surgery , N. Wright , G. Jensen , et al., “Global Initiative for Children’s Surgery: A Model of Global Collaboration to Advance the Surgical Care of Children,” World Journal of Surgery 43, no. 6 (2019): 1416–1425, 10.1007/s00268-018-04887-8. PMC7019676 30623232

[9] C. Okoli and S. D. Pawlowski , “The Delphi Method as a Research Tool: An Example, Design Considerations and Applications,” Information & Management 42, no. 1 (2004): 15–29, 10.1016/j.im.2003.11.002.

[10] I. R. Diamond , R. C. Grant , B. M. Feldman , et al., “Defining Consensus: A Systematic Review Recommends Methodologic Criteria for Reporting of Delphi Studies,” Journal of Clinical Epidemiology 67, no. 4 (2014): 401–409, 10.1016/j.jclinepi.2013.12.002. 24581294

[11] World Bank , World Bank Country Classification Page, (n.d.): (official dataset).

[12] Z. Blumenthal , W. A. Farhat , and K. McQueen , “Enhancing Equitable Impact: Ethical, Legal, and Sustainable Approaches in Short‐Term Surgical Outreach for Global Child Health,” Frontiers in Pediatrics 12 (2025): 1398432, 10.3389/fped.2024.1398432. PMC11758174 39867701

[13] E. Zitzman , H. Berkley , and R. M. Jindal , “Accountability in Global Surgery Missions,” BMJ Global Health 3, no. 6 (2018): e001025, 10.1136/bmjgh-2018-001025. PMC6326286 30687523

[14] I. C. Hageman , P. Midrio , H. J. J. Van Der Steeg , et al., “The European Anorectal Malformation Network (ARM‐Net) Patient Registry: 10‐Year Review of Clinical and Surgical Characteristics,” British Journal of Surgery 111, no. 2 (2024): znae019, 10.1093/bjs/znae019. PMC10870250 38364059

[15] J. Castillo , P. J. Lupo , D. D. Tu , A. J. Agopian , and H. Castillo , “The National Spina Bifida Patient Registry: A Decade’s Journey,” Birth Defects Research 111, no. 14 (2019): 947–957, 10.1002/bdr2.1407. 30403011

