# Supplementary material for: Pediatric Surgeons' Expectations of the EUPSA Global Surgery Committee Across World Bank Income Groups: A Two‐Round Modified Delphi Study
Source: World J Surg. 2026 Sep 1;50(10):2976–86. doi: 10.1002/wjs.70541 (PMC13662516; doi:10.1002/wjs.70541)
Supplement: Supplementary file 1 [file WJS-50-2976-s001.docx]

# Round 2 - Understanding LMIC Paediatric Surgeons' Expectations (EUPSA Global Surgery)

Thank you for taking part in Round 2 of the EUPSA Global Surgery Committee Delphi study.

In this round, you will be asked to prioritise and rate different types of support for paediatric surgery in your country or setting, based on themes identified from Round 1 responses.

Please complete the survey below.

Thank you for your time and contribution.

Based on analysis of the open-ended responses from Round 1, six overarching themes and twenty specific subthemes were identified, reflecting the types of support participants described as important for paediatric surgery across different settings.

In this round, you will be asked to:

1. Rank the six themes according to their importance in your setting
2. Rate the importance of each subtheme using a 1-10 scale
3. Select the three areas you consider top priority for your country or institution

Neutrality note:

These themes and subthemes are derived directly from Round 1 responses. Their order does not imply any predefined priority. Please evaluate each item independently, based on your own experience and local context.

Country of practice

(Please type your country of practice)

Current position/role:	Consultant / Specialist Paediatric Surgeon Consultant / Specialist Paediatric Urologist General Surgeon with paediatric surgery interest Paediatric Surgery/ Paediatric Urology Trainee / Registrar

Fellow (Paediatric Surgery/Urology) Medical Officer or Junior Doctor Other: (free text)

(Please select the option the best applies)

**(This ranking reflects relative priority, not absolute importance. In the next section, you will**

**rate the importance of specific subthemes using a 1-10 scale.)**

**Each rank can be used only once.**

**Please rank these themes in order of priority for paediatric surgery in your setting, where:**

- **1 = highest priority**
- **6 = lowest priority**

**The following six themes were identified from Round 1.**

**Theme prioritisation**

1	2	3	4	5	6

1.

Guidelines, knowledge and educational materials
2.

Training, mentorship, fellowships and exchanges
3.

Research, registries and publications
4.

Advocacy, awareness, workforce and funding
5.

Infrastructure, equipment, telemedicine and service delivery
6. General or cross-cutting

support (broad or

multi-component support)

**The next section asks you to rate more specific types of support within each theme**

For each item below, please rate how important this type of support would be for paediatric surgery in your country or setting, using the following scale:

1 = Not at all important ... 10 = Extremely important.

| **THEME 1: Guidelines, knowledge & educational materials (Non-physical learning resources,**  **knowledge exchange)** | | | | | | | | | |
| --- | --- | --- | --- | --- | --- | --- | --- | --- | --- |
| 1 (Not | 2  | 3  | 4  | 5  | 6  | 7  | 8  | 9 | 10 |
| at all |  |  |  |  |  |  |  |  | (Extrem |
| importa |  |  |  |  |  |  |  |  | ely |
| nt)  |  |  |  |  |  |  |  |  | importa  |
| 1. Standardised clinical |  |  |  |  |  |  |  |  | nt) |

guidelines & protocols

2.

Educational materials and learning resources
3.

Access to expert networks for advice and case discussion

| **THEME 2: Training, mentorship & exchanges (Capacity-building, practical skills development,**  **professional growth)** | | | | | | | | | |
| --- | --- | --- | --- | --- | --- | --- | --- | --- | --- |
| 1 (Not | 2  | 3  | 4  | 5  | 6  | 7  | 8  | 9 | 10 |
| at all |  |  |  |  |  |  |  |  | (Extrem |
| importa |  |  |  |  |  |  |  |  | ely |
| nt)  |  |  |  |  |  |  |  |  | importa  |
| Fellowships or observerships |  |  |  |  |  |  |  |  | nt) |

(local or international)

Hands-on workshops or simulation training

Exchange programmes/twinning between centres or countries

Structured mentorship programmes

| **THEME 3: Research, registries & publications (Data systems, collaborative research, academic**  **support)** | | | | | | | | | |
| --- | --- | --- | --- | --- | --- | --- | --- | --- | --- |
| 1 (Not | 2 | 3 | 4 | 5 | 6 | 7 | 8 | 9 | 10 |
| at all |  |  |  |  |  |  |  |  | (Extrem |
| importa |  |  |  |  |  |  |  |  | ely |
| nt) |  |  |  |  |  |  |  |  | importa |
| Support to establish or |  |  |  |  |  |  |  |  | nt) |

strengthen registries/databases

Opportunities for multicentre collaborative research

Support with publication (writing, fees, journal selection)

Research mentorship (design, statistics, analysis)

| **THEME 4: Advocacy, awareness & workforce (Visibility, policy influence, workforce**  **strengthening)** | | | | | | | | | |
| --- | --- | --- | --- | --- | --- | --- | --- | --- | --- |
| 1 (Not | 2 | 3 | 4 | 5 | 6 | 7 | 8 | 9 | 10 |
| at all |  |  |  |  |  |  |  |  | (Extrem |
| importa |  |  |  |  |  |  |  |  | ely |
| nt) |  |  |  |  |  |  |  |  | importa |
| Fundraising support for |  |  |  |  |  |  |  |  | nt) |

paediatric surgical services

Awareness and

public/health-sector campaigns

Workforce advocacy (posts, training, recognition, remuneration)

Advocacy with ministries and national authorities

| **THEME 5: Infrastructure, equipment & service delivery (Physical resources, system**  **organisation, telemedicine support)** | | | | | | | | | |
| --- | --- | --- | --- | --- | --- | --- | --- | --- | --- |
| 1 (Not | 2 | 3 | 4 | 5 | 6 | 7 | 8 | 9 | 10 |
| at all |  |  |  |  |  |  |  |  | (Extrem |
| importa |  |  |  |  |  |  |  |  | ely |
| nt) |  |  |  |  |  |  |  |  | importa |
| Infrastructure and equipment for |  |  |  |  |  |  |  |  | nt) |

paediatric surgery

Guidance on organising/strengthening paediatric surgical services

Telemedicine or MDT support for complex cases

| **THEME 6: Priority-setting & general support (Cross-cutting support needs, broad or**  **multi-component assistance)** | | | | | | | | | |
| --- | --- | --- | --- | --- | --- | --- | --- | --- | --- |
| 1 (Not | 2 | 3 | 4 | 5 | 6 | 7 | 8 | 9 | 10 |
| at all |  |  |  |  |  |  |  |  | (Extrem |
| importa |  |  |  |  |  |  |  |  | ely |
| nt) |  |  |  |  |  |  |  |  | importa |
| Support for identifying priorities |  |  |  |  |  |  |  |  | nt) |

Comprehensive,

multi-component support packages

If you could prioritise only three items for your	Guidelines - Standardised clinical guidelines & setting, which would they be?	protocols

(Please select up to three.)	Guidelines - Educational materials & learning resources

Guidelines - Access to expert networks

Training - Fellowships/observerships

Training - Hands-on workshops

Training - Exchange programmes

Training - Structured mentorship programmes

Research - Registry/database support

Research - Participation in multicentre research

Research - Publication support (writing, fees, journals)

Research - Research mentorship (design, statistics)

Advocacy - Fundraising support

Advocacy - Awareness campaigns

Advocacy - Workforce advocacy (posts, recognition, remuneration)

Advocacy - Advocacy with ministries/authorities

Infrastructure - Equipment & physical infrastructure

Infrastructure - Service organisation guidance

Infrastructure - Telemedicine / MDT support

General - Support for identifying priorities

General - Comprehensive multi-component support packages

Do you feel that the list of items above adequately	Yes

reflects the needs in your context?	No

If you answered "No", please describe any additional items, types of support, or clarifications you feel

are missing.
