# Supplementary material for: Pediatric Surgeons' Expectations of the EUPSA Global Surgery Committee Across World Bank Income Groups: A Two‐Round Modified Delphi Study
Source: World J Surg. 2026 Sep 1;50(10):2976–86. doi: 10.1002/wjs.70541 (PMC13662516; doi:10.1002/wjs.70541)
Supplement: Supplementary file 2 [file WJS-50-2976-s002.docx]

# Understanding LMIC Paediatric Surgeons' Expectations from the EUPSA Global Surgery Committee - Round 1

Welcome to Round 1 of the EUPSA Global Surgery Delphi Study! Thank you for taking part in this important project.

-

This survey is anonymous. Your answers will not be linked to your identity.

-

At the end, you will have the option to leave an email address only so we can contact you for Round 2 and Round

3. Your email will be stored in a separate database, not connected to your answers.

-

Leaving your email is very important: the Delphi process only works if we can hear your views across all rounds.

-

The study will run in three rounds over several months. Each round should take about 5-10 minutes.

Thank you!

1. Country of practice

(Please type your country of practice)

2. Practice location Urban / City

 Rural

 Both urban and rural practice

(Please select the option the best applies)

3. Which of the following are available at your Dedicated paediatric operating theatre(s)

primary workplace? (select all that apply) Paediatric anaesthesia service (regular)

 Neonatal / Paediatric intensive care unit (NICU / PICU)

 Dedicated paediatric surgical team / unit

 Regular access to imaging (CT / MRI / Ultrasound)

 None of the above

 I am not sure

(Please select all that applies)

4. Current position/role:	Consultant / Specialist Paediatric Surgeon Consultant / Specialist Paediatric Urologist General Surgeon with paediatric surgery interest Paediatric Surgery/ Paediatric Urology Trainee / Registrar

Fellow (Paediatric Surgery/Urology) Medical Officer or Junior Doctor Other: (free text)

(Please select the option the best applies)

If you selected other, please specify

(If you selected other, please specify )

5. Years of experience in paediatric surgery (since	Less than 1 year

first exposure to paediatric surgery):	1-3 years 4-10 years

11-20

More than 20 years

(Please select the option the best applies)

6. Sector of practice: Public sector

 Private sector

 Academic / University hospital

 NGO / Mission hospital

 Other: (free text)

(Please select the option that best applies)

If you selected other, please specify

(If you selected other, please specify )

7. Are you a EUPSA member or have you ever attended a	I am a EUPSA Member

EUPSA Annual Congress? (drop-down)	I am not a EUPSA member, but I have attended a EUPSA conference

 No, I have never attended a EUPSA conference but I am familiar with EUPSA

 No, I have never attended a EUPSA conference and I am not familiar with EUPSA

(Please select the option that best applies)

8. What are up to five things you expect from the EUPSA Global Surgery Committee to support paediatric surgery in your country?

You may share any expectations or suggestions that are important to you: your answers do not need to follow specific categories. However, if helpful, here are

some example areas you might consider (you are welcome to go beyond these):

- Academic development and knowledge sharing - e.g., access to guidelines, educational materials, or expert networks.
- Clinical practice and infrastructure - e.g., improving service delivery, resources, or specialist support.
- Training, mentorship, and capacity building -

e.g., fellowships, workshops, exchange programmes.

- Research support and collaboration - e.g., mentorship, funding opportunities, joint projects.
- Policy and advocacy - e.g., influencing health policies, raising awareness, addressing workforce or funding barriers.
